# Deep Learning–Based Structural Brain Age Estimation in Bipolar Disorder and Schizophrenia: A Single‐Site Pilot Study

**DOI:** 10.1002/hbm.70479

**Published:** 2026-02-19

**Authors:** Akila Weerasekera, Shuqin Zhou, Chao Wang, Zhilang Qiu, Abigail Stein, Margaux Ameer, Virginie‐Anne Chouinard, Ann Shinn, Kathryn E. Lewandowski, Michael Murphy, Mark Halko, Halide Bilge Türközer, Dost Öngür, Fei Du

**Affiliations:** ^1^ Psychotic Disorders Division McLean Hospital Belmont Massachusetts USA; ^2^ McLean Imaging Center McLean Hospital Belmont Massachusetts USA; ^3^ Harvard Medical School Boston Massachusetts USA

**Keywords:** bipolar, brain aging, grad–CAM, schizophrenia

## Abstract

Accelerated brain aging has been implicated in severe mental illnesses, particularly schizophrenia (SZ) and bipolar disorder (BD). Brain–PAD, derived from structural MRI, offers a promising biomarker of neurobiological aging, but its developmental course, within‐group variability, and regional drivers remain incompletely understood. A three‐dimensional convolutional neural network (3D‐CNN) was trained exclusively on healthy controls (HC; *n* = 155) and then applied to independent BD (*n* = 122) and SZ (*n* = 161) groups. Brain–PAD was computed as predicted brain age minus chronological age. Age‐by‐group interactions, within‐group dispersion, and sensitivity analyses (e.g., piecewise regression, inverse probability weighting) were conducted. Gradient‐weighted Class Activation Mapping (Grad–CAM) was used to identify regional contributions to brain age predictions. The 3D‐CNN achieved high accuracy in HC (MAE = 3.05 years, *r* = 0.96), with reduced accuracy in BD (MAE = 8.86, *r* = 0.51) and SZ (MAE = 9.01, *r* = 0.48). Mean Brain–PAD was significantly elevated in BD (+4.2 ± 10.2 years) and SZ (+6.7 ± 8.7 years) relative to HC (+0.7 ± 3.5 years). Age‐by‐group analyses revealed that BD and SZ exhibited elevated Brain–PAD at younger ages, converging toward HC trajectories by midlife, followed by renewed divergence beyond age 40. This pattern was supported by piecewise and spline models showing steeper negative slopes in BD and SZ compared with HC. Variance and quantile regression indicated greater heterogeneity in BD and SZ across the Brain–PAD distribution. Grad–CAM highlighted temporal and frontal regions as central contributors across all groups; in SZ, Brain–PAD correlated positively with whole‐brain (*r* = 0.23, *p* = 0.004), frontal (*r* = 0.21, *p* = 0.009), and temporal (*r* = 0.20, *p* = 0.012) activations, whereas BD showed weaker and more diffuse associations. SZ and BD exhibit elevated Brain–PAD early in adulthood with greater heterogeneity than healthy controls. Frontotemporal regions contribute prominently to brain‐age predictions, reflecting model sensitivity to age‐informative structure. These findings support Brain–PAD as a group‐level marker of apparent brain aging and motivate longitudinal study of midlife divergence.

## Graphical abstract

A deep–learning model (CNN) trained on healthy controls estimated structural brain age in bipolar disorder and schizophrenia. Both disorders showed elevated brain‐predicted age early in adulthood, convergence by midlife, and renewed deviation later, highlighting non‐linear brain aging and frontotemporal regions as key contributors identified by Grad–CAM.

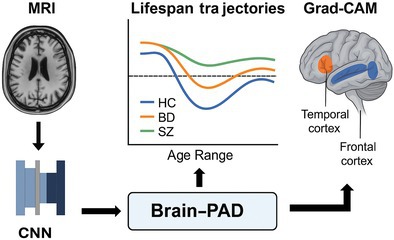

## 1 Introduction

The prediction of brain age from neuroimaging data via machine learning has emerged as a powerful biomarker for quantifying neurobiological aging (Cole 2020). This approach has proven particularly valuable in psychiatry, where significant brain‐predicted age difference (Brain–PAD), indicative of accelerated aging, have been consistently reported in severe mental illnesses such as schizophrenia (SZ) and bipolar disorder (BD) (Kaufmann et al. 2019; Nenadic et al. 2017; Schnack et al. 2016). Converging evidence indicates that these conditions are associated with premature biological aging, as reflected in both peripheral biomarkers (e.g., telomere shortening, epigenetic acceleration) (*Psychosomatic Medicine* 2020; Ayora et al. 2022; McKinney et al. 2018) and neuroimaging measures (Koutsouleris et al. 2014; Hajek et al. 2019).

Structural MRI studies consistently show that individuals with SZ exhibit Brain–PAD values 3–5 years higher than healthy controls (HCs), an effect size exceeding many age‐related neurodegenerative conditions (Kaufmann et al. 2019; Schnack et al. 2016). In BD, findings are more variable, but meta‐analyses indicate moderate Brain–PAD elevations (1–4 years) (Hajek et al. 2019; Van Gestel et al. 2019), particularly in patients with psychotic features or a more severe clinical course. However, emerging evidence highlights the influence of treatment: lithium users show markedly lower Brain–PAD compared with non‐users, with reported differences of 4–5 years, consistent with a potential neuroprotective effect (Van Gestel et al. 2019; van der Markt et al. 2024). These observations align with clinical trajectories of both disorders, where patients often experience age‐associated comorbidities decades earlier than the general population (Rizzo et al. 2014; Kirkpatrick et al. 2014).

The neural substrates of accelerated brain aging appear to diverge between disorders (Kaufmann et al. 2019; Nenadic et al. 2017). In SZ, Brain–PAD correlates strongly with frontotemporal gray matter loss and ventricular enlargement, patterns resembling an exaggerated form of normative aging (Schnack et al. 2016; Vita et al. 2012; van Haren et al. 2011). Longitudinal studies further suggest that these alterations begin early and progress with illness chronicity (Hajek et al. 2019; van Erp et al. 2018). In BD, accelerated aging is less consistent, with reports highlighting limbic (e.g., amygdala, hippocampus) and prefrontal changes (Hajek et al. 2019; Abe et al. 2022; Fries et al. 2020). This heterogeneity may reflect illness subtypes, differential effects of mood episodes, or medication influences such as lithium (Haukvik, Gurholt, Nerland, et al. 2022; Berk et al. 2017; Hajek et al. 2012).

Despite progress, current brain age research faces several challenges (Cole 2020; Liang et al. 2019; Baecker et al. 2021). Traditional machine‐ learning approaches often rely on manually engineered features, which may miss nonlinear and spatially distributed patterns of structural change. Normative modeling is also frequently based on mixed patient–control data sets, raising the risk of contamination from disease‐related variance (Rutherford et al. 2022). Moreover, most studies emphasize global brain age metrics, offering limited insight into the specific regions that drive accelerated aging in SZ and BD.

Recent work has demonstrated that convolutional neural networks (CNN) can be used to estimate brain age from structural MRI while providing neuroanatomic attribution maps that link model predictions to regional brain features and cognitive or clinical measures (Yin et al. 2023, 2025). In parallel, a growing literature has developed machine learning approaches for brain‐age prediction, bias correction, and forecasting of age‐related trajectories across the lifespan (Liu et al. 2025; Cheng et al. 2025). These studies have established the feasibility of interpretable brain‐age modeling and highlighted both the promise and limitations of such approaches.

In this study, we applied a three‐dimensional convolutional neural network (3D‐CNN) to estimate brain age from structural MRI, following established deep‐learning approaches for brain‐age prediction. The model was trained exclusively on HC data to derive normative aging estimates, and Brain–PAD was subsequently evaluated in individuals with SZ and BD. Consistent with prior work incorporating attribution mapping in brain‐age models, Gradient‐weighted Class Activation Mapping (Grad–CAM) was used as a post hoc interpretability tool to visualize regions contributing most strongly to model predictions. Importantly, these attribution maps were used to characterize model sensitivity to age‐informative features rather than to infer disorder‐specific anatomical mechanisms or localized biological drivers of accelerated aging.

As a pilot study, we examined a single‐site data set of 438 participants, including 122 BD patients, 161 SZ patients, and 155 HCs, all recruited, imaged, and processed within a harmonized pipeline. We hypothesized that SZ and BD would show elevated Brain–PAD relative to HCs, with greater acceleration in SZ, and that Grad–CAM would reveal disorder‐specific regional contributions, with frontotemporal regions predominating in SZ and limbic–prefrontal regions in BD. In addition, we tested whether age‐related divergence in Brain–PAD trajectories would be observed, such that patients begin near‐normal but deviate increasingly with age, particularly after midlife and whether BD and SZ would differ in the steepness or heterogeneity of these slopes. This work provides a proof‐of‐concept demonstration of CNN‐based normative modeling in severe mental illness. By relying on minimally processed T1‐weighted MRI data, our approach establishes a streamlined framework for deriving brain‐age metrics without reliance on extensive preprocessing pipelines. Importantly, we apply Grad–CAM, previously used in interpretable brain‐age modeling, to examine regional features associated with Brain–PAD in SZ and BD. Despite its smaller scale, this study highlights both the feasibility and clinical potential of deep learning–derived brain age metrics, laying the groundwork for replication in larger, multi‐site cohorts.

## 2 Methods

### 2.1 Data Selection

This study utilized neuroimaging and demographic data from patients with SZ, BD, and HCs, obtained from the McLean Psychotic Disorders Division Clinical Data Repository. The data set comprised 438 participants, including 122 BD patients (69 males, 53 females; mean age 32 ± 9 years), 161 SZ patients (108 males, 53 females; mean age 30 ± 9 years; first‐episode and chronic), and 155 healthy controls (73 males, 82 females; mean age 29 ± 9 years). Demographic and clinical characteristics are summarized in Tables 1, 2 and Figures S1–S2. In this study, all scans were acquired using two 3 T clinical MRI scanner models (Siemens Trio and Siemens MAGNETOM Prisma) with a standardized 3D MPRAGE sequence (TR = 1900 ms; TE = 2.26 ms; FOV = 250 mm; voxel dimensions = 1.0 × 1.0 × 1.0 mm^3^; 176 slices.).

**TABLE 1:** MRI scanner parameters and participant demographics.

Scan parameters	Siemens magnetom prisma	Siemens tim trio
Field strength	3 T	3 T
Head coil	64‐channel	32‐channel
Sequence	T1‐weighted MPRAGE	T1‐weighted MPRAGE
Voxel size (mm^3^)	1.0 × 1.0 × 1.0	1.0 × 1.0 × 1.0
TR (ms)	2530	1900
TE (ms)	1.69	2.26
FOV (mm)	256	250
Number of slices	176	176
Total participants (N)	176	262
HC	77 (35 M, 42 F); Age: 28 ± 8 years	78 (38 M, 40 F); Age: 29 ± 9 years
SZ	79 (44 M, 35 F); Age: 35 ± 9 years	82 (49 M, 33 F); Age: 35 ± 8 years
BD	20 (12 M, 8 F); Age: 34 ± 9 years	102 (58 M, 44 F); Age: 33 ± 9 years

**TABLE 2:** PANSS scores and disease duration by diagnosis group.

Group	PANSS positive	PANSS negative	PANSS general	Disease duration (years)
BD (*N* = 122)	15.83 ± 8.25	9.94 ± 3.13	27.55 ± 8.29	7.6 ± 8.41
SZ (*N* = 161)	17.02 ± 6.98	15.23 ± 6.98	31.47 ± 9.24	10.4 ± 9.2

*Note:* Values are presented as mean ± SD.

### 2.2 MRI Image Preprocessing

The T1‐MPRAGE images were processed using FreeSurfer's automated pipeline (version 7.2) (Henschel et al. 2020) to extract neuroanatomical features. First, the raw images underwent motion correction to minimize artifacts from head movement, resulting in the motion‐corrected volume. Next, intensity normalization was applied to correct for scanner‐related bias fields, producing the bias‐corrected image. The skull‐stripping step then isolated brain tissue from non‐brain structures (e.g., skull, neck) using a watershed algorithm, generating the final skull‐stripped brain image. All outputs were visually inspected to ensure processing accuracy.

All T1‐weighted images were further preprocessed prior to model input. Native 256 × 256 × 256 volumetric images were isotropically downsampled to 128 × 128 × 128 voxels using trilinear interpolation (PyTorch's F. interpolate with mode = “trilinear”) to optimize computational efficiency while preserving structural information. Images underwent intensity normalization through: (1) clipping at the 1st and 99th percentiles, (2) *z*‐scoring within each volume (mean = 0, SD = 1), and (3) age normalization across the training set using StandardScaler.

### 2.3 Deep Learning Architecture for Brain Age Prediction

#### 2.3.1 Convolutional Neural Network (CNN) Architecture

**FIGURE 1:**
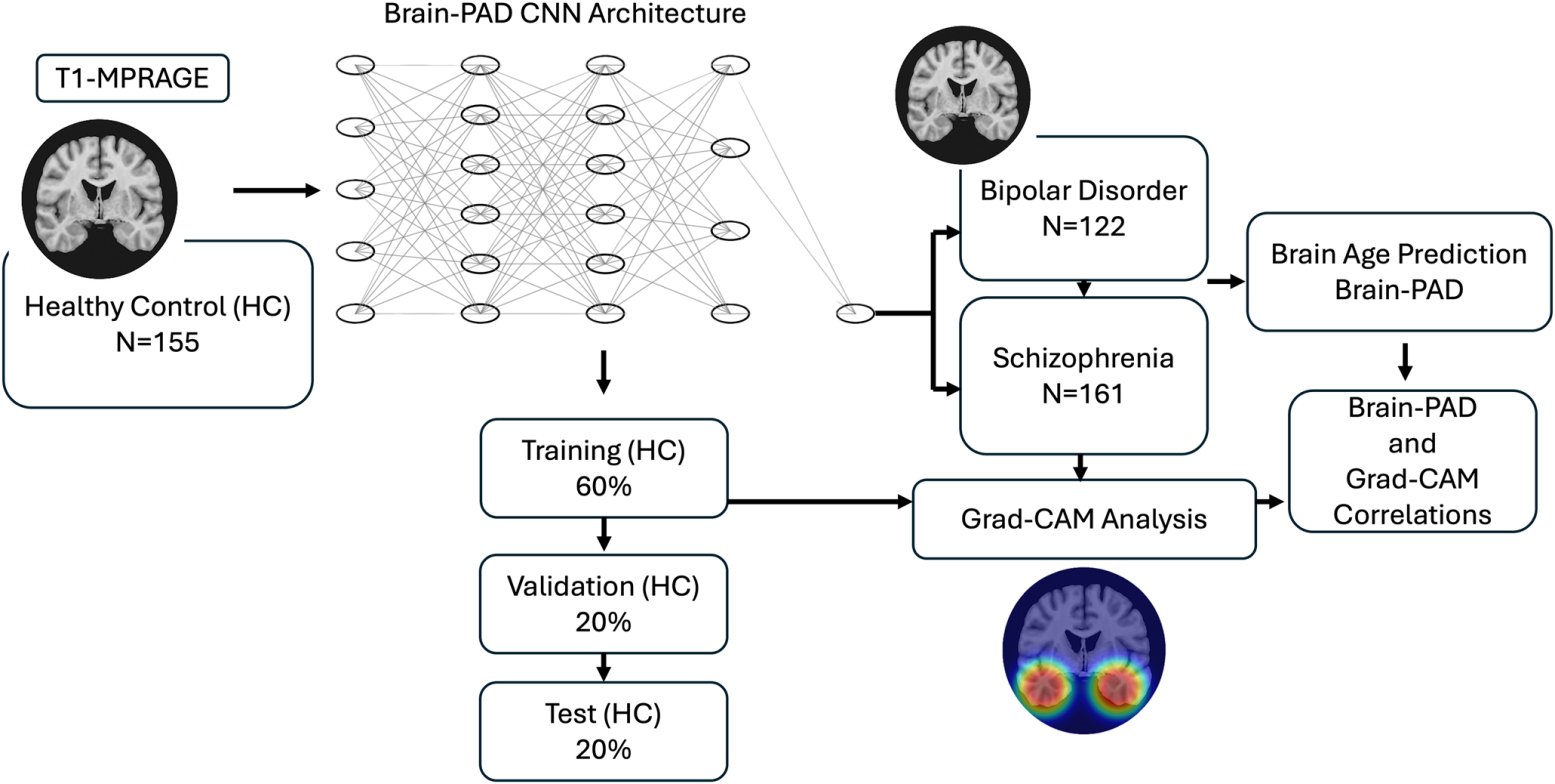
Schematic overview of the Brain–PAD prediction framework. Structural T1‐weighted MRI images from HCs were used to train a 3D‐CNN to predict chronological age. The trained model was then applied to independent data sets of BD and SZ patients to generate predicted brain ages. Brain–PAD values were calculated as the difference between predicted and chronological age. Grad–CAM analysis was subsequently used to identify regional contributions driving model predictions and correlate with brain–PAD.

We implemented a 3D‐CNN (Figure 1) with five sequential 3D convolutional blocks. Each block consisted of a 3 × 3 × 3 convolution with same padding, batch normalization, rectified linear unit (ReLU) activation, and 2 × 2 × 2 max pooling (stride = 2). Channel depth increased progressively across layers (16 → 32 → 64 → 128 → 256). The fully connected head comprised a 512‐unit dense layer with ReLU activation, dropout (*p* = 0.1), and a linear output layer (1 unit).

#### 2.3.2 Covariate Integration

The architecture incorporated a dedicated branch for categorical covariates (sex and scanner type). Scanner type was modeled using feature‐wise linear modulation (FiLM) layers to condition convolutional filters, while sex‐stratified batch normalization layers were implemented to account for sex‐related effects.

#### 2.3.3 Training Protocol

The network was optimized using AdamW (learning rate = 1 × 10^−4^; weight decay = 1 × 10^−5^) with Huber loss, balancing L1 and L2 sensitivity. Regularization included dropout (*p* = 0.1) and gradient clipping (max_norm = 1.0). Data augmentation consisted of random 3D affine transformations (±5 rotation, ±5% translation, ±10% scaling) and horizontal flipping (*p* = 0.5). Training was performed with a batch size of 32 for a maximum of 150 epochs, with early stopping (based on validation loss) and adaptive learning rate reduction (patience = 5 epochs).

#### 2.3.4 Data Partitioning

HCs were randomly partitioned into training (60%), validation (20%), and test (20%) sets. BD and SZ participants were retained as separate held‐out groups for independent evaluation.

#### 2.3.5 Implementation Details

All models were implemented in PyTorch with CUDA acceleration where available. Reproducibility was ensured by fixing random seeds, saving model checkpoints, and tracking training curves. Grad–CAM visualizations were generated from the fourth convolutional block (conv4) to aid interpretability.

#### 2.3.6 Model Performance Evaluation

Model accuracy was quantified on independent test samples from HCs and patient groups using mean absolute error (MAE), Pearson's correlation coefficient (r), and the coefficient of determination (*R* ^*2*^). Predictions for patient groups were used to compute Brain–PAD values (predicted age − chronological age).

### 2.4 Age‐By‐Group Divergence in Brain–PAD (HC, BD, SZ)

We modeled brain‐predicted age difference (Brain–PAD) as a function of age and diagnosis to test whether BD and SZ begin near HC but diverge with age. The primary analysis used a linear interaction model (Brain–PAD ~ age × group, covarying sex and scanner type), with core inference on the age × group slopes versus HC and a direct BD–SZ contrast. To safeguard against model misspecification, we complemented this with spline‐ and piecewise‐age regressions, which formally tested whether divergence was concentrated in midlife. For visualization, we also plotted LOESS smooths (locally estimated scatterplot smoothing), a nonparametric method that flexibly captures nonlinear trends without assuming a global functional form. Formal inference, however, relied on spline and piecewise models. Heterogeneity was evaluated using variance tests and quantile regression, and sensitivity analyses (age trimming, inverse probability weighting, leave‐one‐out influence) were conducted. Robustness was further assessed with covariate adjustment and robust regression.

### 2.5 Brain–PAD and Grad–CAM Correlations

Associations between Brain–PAD and mean Grad–CAM activation values were examined using Pearson's correlation coefficients, calculated separately within each diagnostic group (HC, BD, SZ) and across the pooled cohort. Regression lines with 95% confidence intervals were plotted for visualization. To control for potential confounds, partial correlations were performed with age, sex, and scanner type as covariates.

### 2.6 Multiple Comparisons

False discovery rate (FDR) correction (Benjamini–Hochberg procedure) was applied to sets of regional correlation analyses within each group. FDR‐adjusted *p* values are reported, with *p* < 0.05 considered statistically significant.

### 2.7 Software

Analyses were conducted in Python (v3.9) using pandas, numpy, nilearn, and statsmodels, with visualizations generated using matplotlib, seaborn, and GraphPad Prism v10.

## 3 Results

### 3.1 Model Performance and Brain–PAD Estimation in All Groups

**FIGURE 2:**
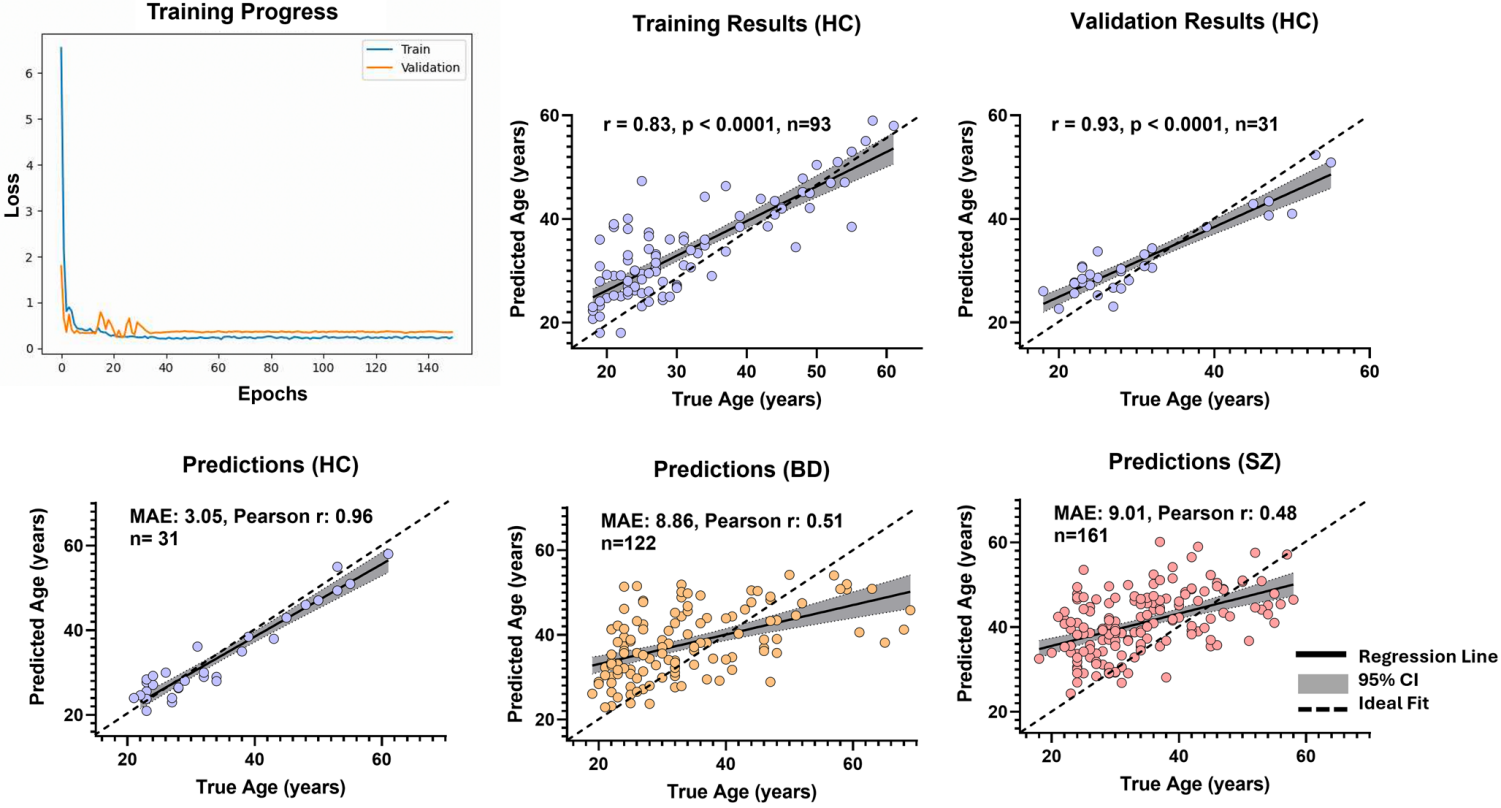
Training performance and brain‐age prediction accuracy across groups. (Top left) Training and validation loss curves across 150 epochs demonstrate convergence of the convolutional neural network model. (Top middle and right) Training and validation results for HCs show significant correlations between true and predicted age (training: *R* = 0.83, *p* < 0.0001; validation: *R* = 0.71, *p* < 0.0001). (Bottom row) Predicted versus true chronological age in independent test sets for HCs, BD, and SZ. The model achieved the highest accuracy in HCs (MAE = 3.05 years, Pearson *r* = 0.96), with reduced accuracy in BD (MAE = 8.86 years, Pearson *r* = 0.51) and SZ (MAE = 9.01 years, Pearson *r* = 0.48). *Brain–PAD is calculated as predicted age minus chronological age. A positive value indicates an “older” appearing brain. Abbreviations: MAE, mean absolute error; Brain–PAD, Brain‐Predicted Age Difference; r, Pearson coefficient; SD, standard deviation. In each scatterplot, the solid black line represents the regression fit, the shaded gray area the 95% confidence interval (CI), and the dashed diagonal line denotes the line of identity, where the predicated age equals the true age.

Our CNN model demonstrated stable and consistent learning, with both training and validation loss steadily decreasing and converging toward zero over 150 epochs (Figure 2, top left). This indicates effective model optimization without evidence of overfitting.

In HC, prediction accuracy was high, with a mean absolute error (MAE) of 3.05 years, Pearson correlation coefficient (*r*) of 0.96, and *R* ^*2*^ of 0.90 (Figure 2, bottom left). Training and validation subsets of HC also showed strong associations between predicted and true age (training: *r* = 0.83, *p* < 0.0001, *n* = 93; validation: *r* = 0.93, *p* < 0.0001, *n* = 31; Figure 2, top middle and top right), confirming the reliability of the model in normative data.

By contrast, model performance was reduced in the patient groups (Figure 2, bottom middle and bottom right). In BD, the model yielded an MAE of 8.86 years (*r* = 0.51, *R* ^*2*^ = 0.23), while in SZ, prediction error was similar (MAE = 9.01 years) with a modest correlation (*r* = 0.48, *R* ^*2*^ = 0.23). Across groups (Table 3), Brain–PAD was elevated, with a mean of + 4.18 years in BD and + 6.73 years in SZ, compared with + 0.65 years in HC. Together, these findings indicate that the CNN accurately captures normative aging trajectories but detects systematic deviations in BD and SZ consistent with elevated Brain–PAD.

**TABLE 3:** Brain age estimation metrics across HC, BD, and SZ groups.

Group	MAE	Pearson's *r*	*R* ^*2*^	Brain–PAD mean ± SD (years)
HC	3.05	0.96	0.92	0.65 ± 3.49
BD	8.86	0.51	0.26	4.18 ± 10.22
SZ	9.01	0.48	0.23	6.73 ± 8.65

Abbreviation: MAE: mean absolute error.

**FIGURE 3:**
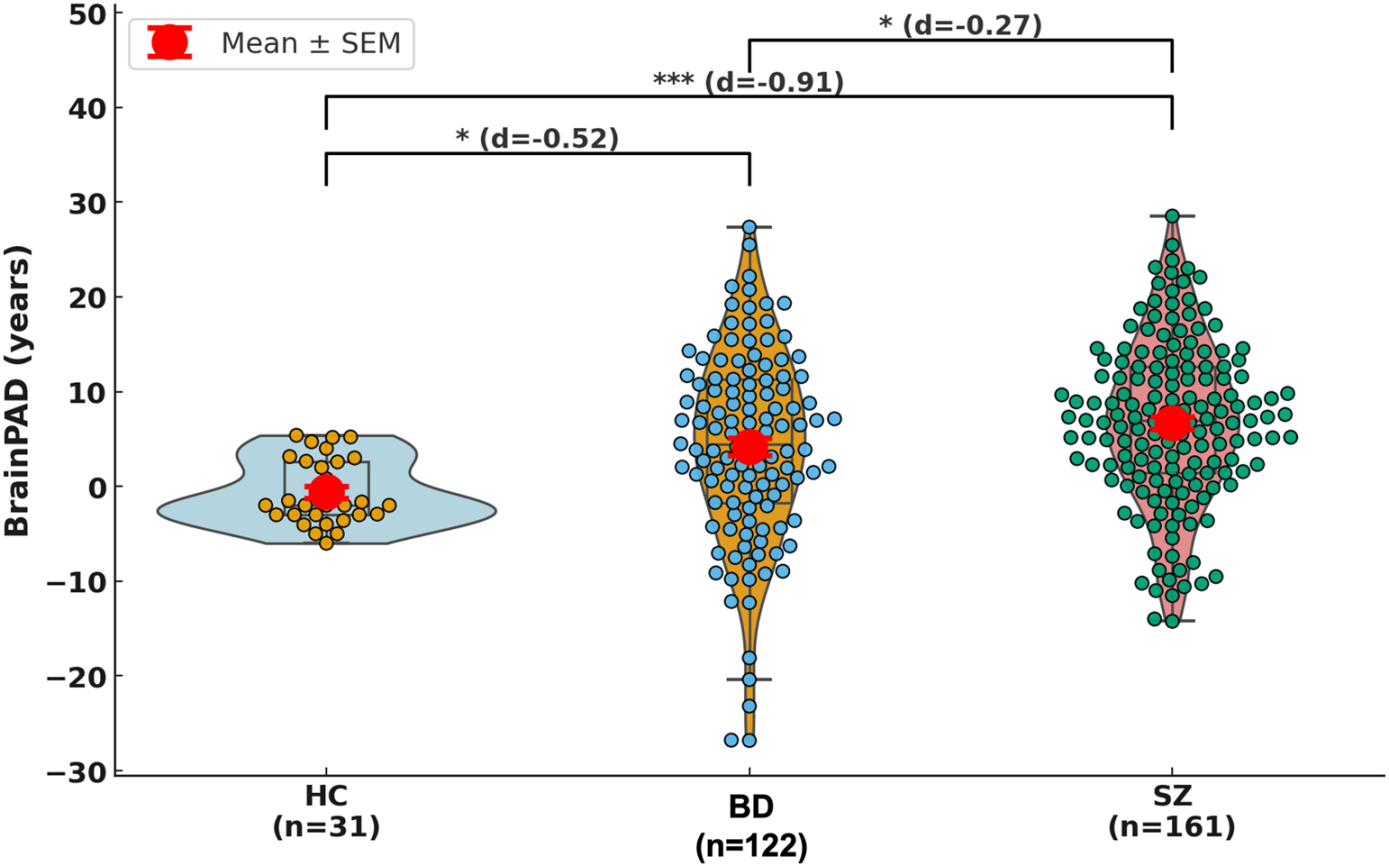
Brain–PAD distributions across groups. Raincloud plots depict brain‐predicted age difference (Brain–PAD, years) in HCs (light blue), BD (orange), and SZ (light red). Each distribution combines a violin (probability density), boxplot (median and interquartile range), and individual subject data points (circles). Group means ± standard error of the mean (SEM) is highlighted in bold red markers with error bars. Horizontal brackets indicate pairwise group comparisons based on Tukey's HSD post hoc tests; asterisks denote significance levels (*p < 0.05, **p < 0.01, ***p < 0.001), and effect sizes (Cohen's d) are reported in parentheses.

The raincloud/violin plot further illustrates group differences in Brain–PAD (Figure 3). Statistical comparisons confirmed significant group differences: BD and SZ each differed from HC (Cohen's *d* = −0.52 and −0.91, respectively), and BD and SZ also differed modestly from each other (*d* = −0.27). Importantly, the wider spread of values in BD and SZ highlights increased interindividual heterogeneity relative to HC. These results reinforce that accelerated brain aging is a robust group‐level feature of both disorders, with particularly strong effects in SZ.

### 3.2 Brain–PAD Trajectories Across Age by Group

**FIGURE 4:**
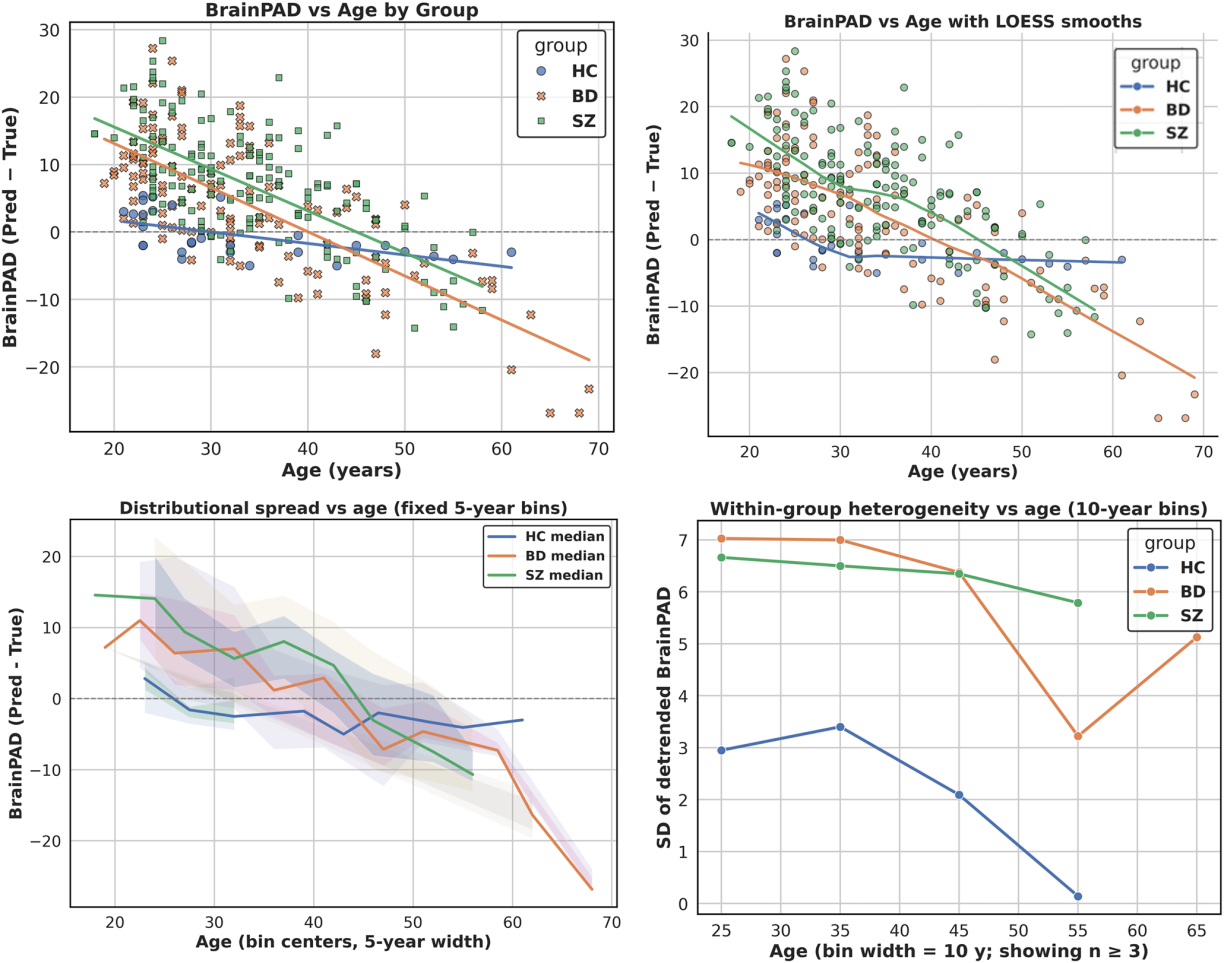
Age‐by‐Group Divergence in Brain–PAD. (Top Left) Scatterplot of Brain–PAD versus age with group‐wise linear regression fits for HC, BD, and SZ. (Top Right) Same scatterplot with LOESS smooths, shown for illustration of potential non‐linear trajectories across the age range. (Bottom Left) Ribbon plot depicting age‐stratified distributions of Brain–PAD by 5‐year bins. Lines represent group‐wise medians; shaded bands denote interquartile range (darker) and 10%–90% range (lighter), highlighting consistent divergence across the distribution. (Bottom Right) Within‐group heterogeneity in Brain–PAD, indexed as the standard deviation of residuals after detrending by age, plotted across 10‐year bins. Both BD and SZ show greater dispersion compared with HC, particularly after midlife. Together, these panels demonstrate that while BD and SZ begin with Brain–PAD values similar to HC in youth, they progressively diverge with age, most markedly in midlife and beyond.

Visual inspection of scatterplots and LOESS smooths suggested that BD and SZ exhibited elevated Brain–PAD at younger ages, converging toward HC trajectories by midlife, followed by renewed divergence beyond age 40 (Figure 4, top row). The LOESS curves were included for visualization of potential nonlinear trajectories; all formal statistical inference relied on the spline and piecewise models described in the Methods.

A linear model testing Brain–PAD ~ Group × True Age confirmed these visual trends (Table S1). Relative to HC, both BD (*β* = +22.07, *t* = 5.60, *p* < 0.001) and SZ (*β* = +23.87, *t* = 5.96, *p* < 0.001) showed significantly higher Brain–PAD at younger ages. The group × age interactions were negative and highly significant for both BD (*β* = −0.51, *t* = −4.63, *p* < 0.001) and SZ (*β* = −0.48, *t* = −4.26, *p* < 0.001), indicating steeper age‐related decline relative to HC, that is, an early elevation in Brain–PAD followed by age‐related normalization.

Piecewise regression with a knot at 40 years further revealed that pre‐40 slopes were nonsignificant, whereas post‐40 slopes were markedly more negative in BD (*β* = −0.539, *p* = 0.034) and SZ (*β* = −0.624, *p* = 0.016), consistent with apparent late‐life divergence in cross‐sectional Brain–PAD estimates. Direct BD–SZ contrasts showed no slope differences (*χ* ^2^ = 0.20–0.77, n.s.).

Beyond mean slopes, heterogeneity analyses demonstrated greater within‐group dispersion in BD and SZ relative to HC (Brown–Forsythe W = 10.71, *p* = 3.2 × 10^−5^), evident both before age 40 and at older ages (Figure 4, bottom right). Quantile regression confirmed that age effects were broadly distributed across Brain–PAD percentiles (*τ* = 0.5: BD *β* = −0.453, *p* = 0.005; SZ *β* = −0.484, *p* = 0.003), indicating that these patterns were not driven by a small subset of individuals. Ribbon plots of age‐stratified medians and dispersion (Figure 4, bottom left) further illustrated this distributed effect. Sensitivity analyses (age trimming, inverse probability weighting, and robust regression) yielded similar or stronger age‐related trends. Spline models revealed non‐linear, biphasic trajectories characterized by apparent convergence toward HC in early‐to‐mid adulthood and divergence at later ages; however, given the cross‐sectional design, uneven age distributions, and known biases in brain‐age estimation, these later‐life patterns should be interpreted cautiously and viewed as descriptive rather than indicative of true late‐life acceleration.

Together, these convergent results indicate that BD and SZ begin with elevated Brain–PAD compared with HC in youth, converge by midlife, and diverge again in later adulthood, reflecting apparent age‐related deviation and non‐linear brain‐aging trajectories in both patient groups.

### 3.3 Grad–CAM Activation

**FIGURE 5:**
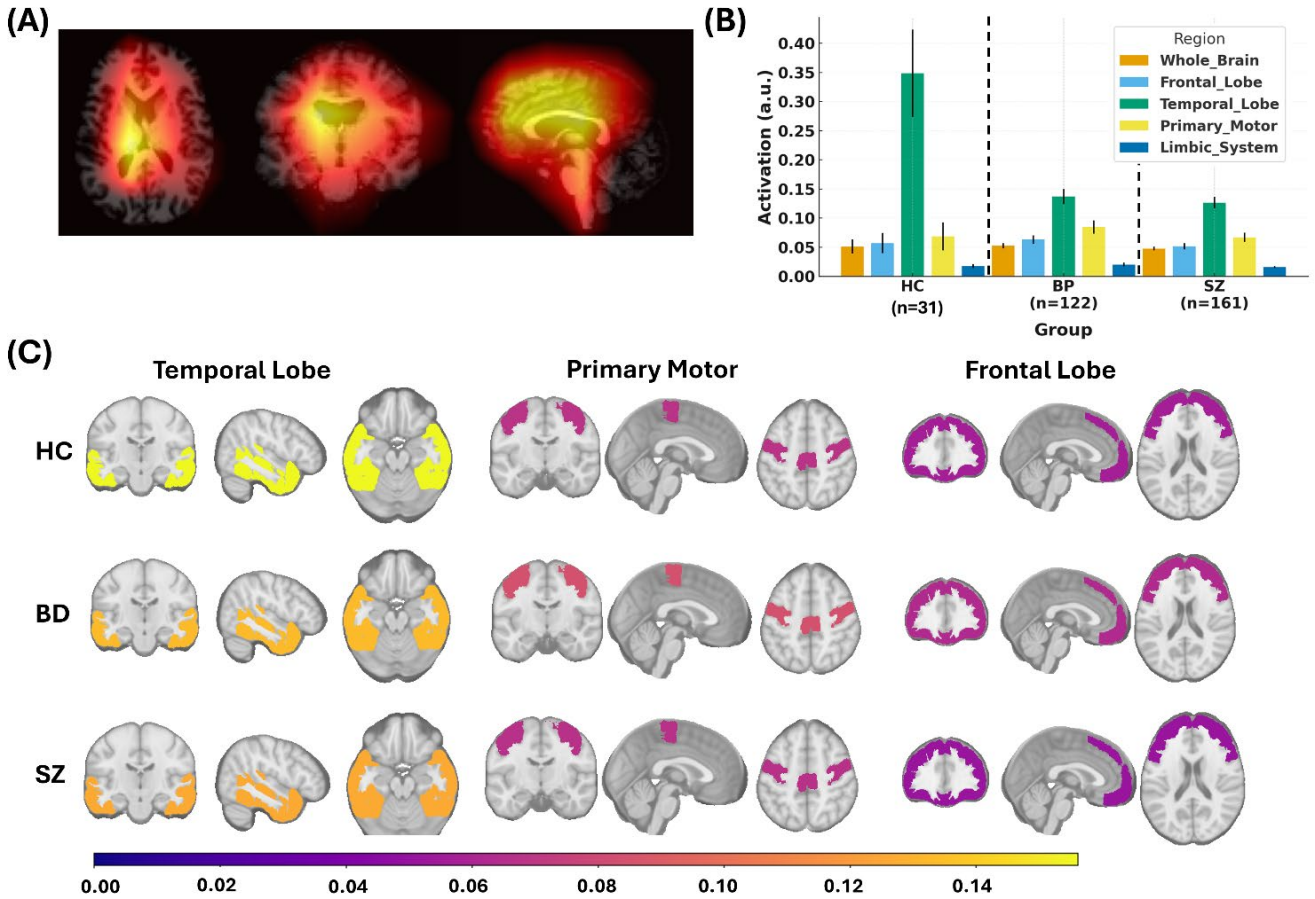
Regional Grad–CAM activation patterns underlying Brain–PAD. (A) Representative mean Grad–CAM map on a T1‐weighted image HC group. (B) Group‐wise mean activation across key regions, with emphasis on the frontal lobe, temporal lobe, primary motor cortex, and limbic system (other regions assessed included whole brain, occipital lobe, primary sensory cortex, and cerebellar lobe). (C) Group‐average Grad–CAM overlays in temporal, motor, and frontal regions, shown for HC (top), BD (middle), and SZ (bottom) on the MNI152 T1 template. Columns display coronal, sagittal, and axial views. Warmer colors indicate stronger activations, while cooler colors indicate weaker activations, highlighting both shared and diagnosis‐specific contributions to brain age estimates. Together, these panels show that temporal and frontal regions disproportionately drive Brain–PAD in SZ, whereas BD exhibits more variable and less regionally specific patterns.

Grad–CAM analyses were used as an exploratory interpretability tool to visualize regions contributing most strongly to Brain–PAD predictions. Importantly, Grad–CAM reflects model sensitivity to input features rather than direct neurobiological aging or anatomical causation. As such, highlighted regions should not be interpreted as localized biological drivers of apparent brain aging, particularly given the limited statistical significance of regional effects after correction. We examined regional Grad–CAM activation patterns across HC, BD, and SZ (Figure 5). As shown in the representative overlays (Figure 5A,C), model attention localized primarily to frontal and temporal cortices, with additional contributions from primary motor regions. Group‐wise mean activations (Figure 5B) revealed that whole‐brain values were comparable across groups (HC: 0.051 ± 0.044; BD: 0.053 ± 0.048; SZ: 0.048 ± 0.039; ANOVA *p* > 0.1). Frontal activations were slightly higher in BD (0.063 ± 0.080) compared with SZ (0.051 ± 0.065) and HC (0.057 ± 0.057), though this difference did not reach significance (*p* = 0.12). Temporal lobe activations were highest in HC (0.342 ± 0.169) relative to BD (0.134 ± 0.131) and SZ (0.127 ± 0.127), with a trend toward reduced temporal signal in patient groups (*p* = 0.09). Parietal and limbic activations were low across groups, with BD showing modest but nonsignificant increases. Primary motor and sensory cortices exhibited modest activations across all groups. Grad–CAM maps also showed notable ventricular contributions, consistent with the strong age sensitivity of ventricular expansion and global atrophy in brain‐age models (results not shown). Overall, the Grad–CAM analyses (Figure 5) highlighted consistent attention to temporal and frontal regions across groups, with a trend toward reduced temporal lobe contributions in BD and SZ and slightly higher frontal/parietal activations in BD. None of these differences survived multiple‐comparison correction, underscoring the need for larger samples to clarify regional specificity.

### 3.4 Regional Grad–CAM Activation and Brain–PAD Associations

**FIGURE 6:**
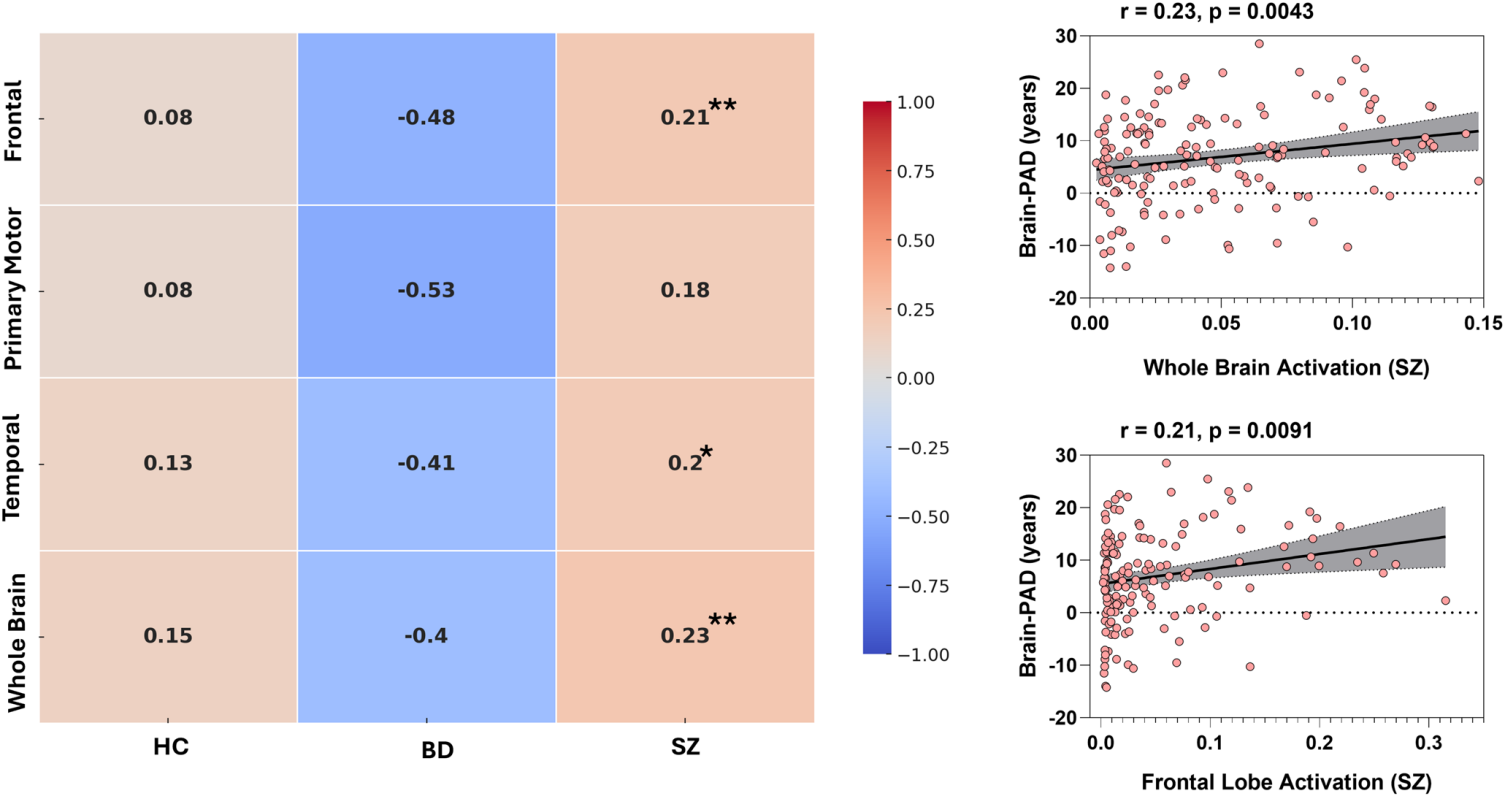
Correlations between Brain–PAD and regional Grad–CAM activations. (Left) Heatmap showing Pearson correlation coefficients between Brain–PAD and mean Grad–CAM activation across frontal, primary motor, temporal, and whole brain regions, separately for HC, BD, and SZ. Warm colors represent positive correlations, and cool colors represent negative correlations. Notably, BD showed negative associations between Brain–PAD and regional activations, whereas SZ showed consistent positive associations. (Right) Scatterplots illustrating significant positive correlations in SZ between Brain–PAD and whole‐brain activation (*r* = 0.23, *p* = 0.0043) and between Brain–PAD and frontal lobe activation (*r* = 0.21, *p* = 0.0091). Shaded regions represent 95% confidence intervals for the regression line. Asterisks indicate statistical significance (*p < 0.05; **p < 0.01, FDR‐corrected).

Grad–CAM analyses revealed that higher Brain–PAD was associated with greater CNN attention to temporal and frontal cortices as well as whole‐brain activation. Across the full sample, Brain–PAD correlated positively with whole‐brain activation (*r* = 0.17, *p* = 0.004), the temporal lobe (*r* = 0.15, *p* = 0.013), and the frontal lobe (*r* = 0.13, *p* = 0.035), with a trend‐level effect in the primary motor cortex (*r* = 0.12, *p* = 0.058) and no significant association in the parietal lobe (*r* = 0.06, *p* = 0.377) (Table 4). Group‐stratified analyses showed that these effects were driven primarily by SZ (Figure 6), where Brain–PAD was robustly and positively related to whole‐brain (*r* = 0.23, *p* = 0.004), frontal (*r* = 0.21, *p* = 0.009), and temporal (*r* = 0.20, *p* = 0.012) activations, with the motor cortex also showing a moderate positive association. By contrast, no regional correlations reached significance in BD, consistent with more diffuse or heterogeneous contributions, while HC showed uniformly weak and nonsignificant associations, reflecting the expected stability of predictions in the normative training cohort. Taken together, these findings identify frontal and temporal cortices as key regional drivers of age‐related deviations in SZ, whereas elevated Brain–PAD in BD was less regionally specific.

**TABLE 4:** Associations of regional GRAD–CAM activation with brain–PAD across HC, BD, and SZ.

Region	Pearson *r*	*p*
Whole brain	0.17	0.0044
Temporal lobe	0.15	0.0134
Frontal lobe	0.13	0.0351
Primary motor	0.12	0.0583
Parietal lobe	0.06	0.3770

## 4 Discussion

In this single‐site pilot study, we applied a CNN‐based normative modeling framework to examine apparent structural brain aging in SZ and BD. Both patient groups exhibited significantly elevated Brain–PAD relative to HCs, with larger deviations observed in SZ (mean + 6.7 years) than in BD (mean + 4.2 years). These findings are consistent with prior meta‐analyses reporting Brain–PAD elevations of ~ + 3 years in severe mental illness, with the largest effects in SZ (Blake et al. 2023; Constantinides et al. 2023; Lee et al. 2021; Xi et al. 2022; Joo et al. 2025). In contrast, Brain–PAD findings in BD are inconsistent: while some smaller studies report no differences from controls (Nenadic et al. 2017; Shahab et al. 2019), larger studies have found moderate elevations ranging from 0.3 to 4.28 years (Kaufmann et al. 2019; Van Gestel et al. 2019; de Nooij et al. 2019; Tonnesen et al. 2020). Our results are consistent with the latter interpretation, suggesting that BD, similar to SZ, is associated with elevated Brain–PAD relative to healthy controls, albeit with smaller effect sizes. Importantly, our harmonized, single‐site data set processed through a uniform pipeline minimizes methodological heterogeneity and strengthens this inference.

Beyond mean differences, we observed pronounced age‐related trajectory effects. Both BD and SZ exhibited elevated Brain–PAD at younger ages relative to HC, indicating older‐appearing brains early in adulthood. Their trajectories then converged toward HC by midlife, followed by renewed divergence beyond age 40. This biphasic pattern was robust across linear, piecewise, and spline models, with sensitivity analyses (age trimming, inverse probability weighting, robust regression) yielding consistent or stronger effects.

Within‐group heterogeneity was also greater in BD and SZ than in HC, evident across both younger and older participants. Quantile regression confirmed that steeper negative slopes were present across lower, median, and upper quantiles, indicating that age‐related differences in Brain–PAD were broadly distributed across individuals rather than driven by outliers. This combination of elevated group‐level Brain–PAD and heightened variability is consistent with models proposing that severe mental illness is associated with greater deviation from normative brain structure, potentially reflecting the cumulative impact of biological burdens such as allostatic load, oxidative stress, and metabolic dysregulation (Juster et al. 2010; Flatow et al. 2013; Brown et al. 2014). For BD in particular, treatment history may be an additional factor. Prior work has shown that lithium use is associated with markedly smaller Brain–PAD than non‐use (Van Gestel et al. 2019; van der Markt et al. 2024), and the diffuse, heterogeneous patterns we observed may partly reflect variability in medication exposure, although we lacked detailed lithium histories to test this directly.

Interpretability analyses further localized age‐informative signals, with Grad–CAM maps highlighting temporal and frontal cortices, alongside contributions from primary motor regions and whole‐brain activation. These regions are also among the most vulnerable to normative aging, with robust age‐related thinning and atrophy linked to declines in executive and memory functions (Raz et al. 2005; Salat et al. 2004; Fjell and Walhovd 2010) Our findings suggest that SZ may represent an exaggerated manifestation of these normative trajectories, consistent with prior evidence of accelerated frontotemporal aging in psychosis (Schnack et al. 2016) BD, by contrast, showed weaker and more diffuse regional associations, potentially reflecting neurobiological heterogeneity and treatment effects.

Our observation that BD and SZ groups exhibited elevated Brain–PAD already in early adulthood suggests that neurodevelopmental or early disease processes may contribute to premature structural aging before midlife divergence occurs. Several recent studies support this interpretation. For example, Haukvik et al. (2022) reported that individuals with first‐episode SZ exhibited significantly higher Brain–PAD (+3 years) compared with controls, whereas individuals in the early or at‐risk stages of BD showed no Brain–PAD difference. This finding suggests that SZ may involve earlier neurodevelopmental deviation, while BD follows a more normative trajectory initially. However, more recent longitudinal evidence indicates that early brain–PAD elevation can also occur in bipolar‐spectrum disorders, even during first‐episode mania (FEM). In a deep‐learning study of youth with FEM (ages 15–25), Han et al. (2025) found that participants had significantly higher baseline Brain–PAD (+2 years) compared with controls, but that this elevation remained stable over 12 months regardless of lithium or quetiapine treatment. This suggests that the early structural offset may represent a trait‐like neurodevelopmental alteration rather than progressive degeneration in the short term. Consistent with these observations, meta‐analytic and cross‐sectional studies have shown elevated Brain–PAD in chronic SZ and, to a lesser extent, BD (Nenadic et al. 2017; Schnack and Kahn 2016). The early deviations observed in our study may likely reflect the combination of neurodevelopmental vulnerability (Lewis and Levitt 2002; Fatemi and Folsom 2009) and early‐onset disease‐related processes, such as aberrant synaptic pruning (Sekar et al. 2016), oxidative stress (Steullet et al. 2017; Fraguas et al. 2019) or neuroinflammation (Muller 2018; Najjar and Pearlman 2015) that precede the more pronounced midlife divergence seen in chronic illness.

Nevertheless, our finding that group divergence was most pronounced in midlife aligns with broader evidence that this period represents a critical window for accelerated neurobiological aging in severe mental illness. By the fourth and fifth decades of life, patients with BD and SZ often accumulate greater illness duration, relapse burden, and medication exposure, alongside heightened rates of metabolic and cardiovascular comorbidities. These factors converge with chronic stress, inflammatory, and oxidative processes to amplify structural brain decline in frontal and temporal cortices (Kaufmann et al. 2019; Frangou et al. 2022). Midlife thus appears to mark the point at which cumulative biological and clinical load translates into steeper Brain–PAD slopes, helping to explain why group trajectories diverge most strongly from healthy controls during this period.

Together, these findings suggest that apparent brain aging is associated with alterations in neural circuits involved in salience processing and cognitive control. While dopaminergic and circuit‐level mechanisms provide one plausible explanation, other biological pathways may also contribute. In support of a neuroinflammatory hypothesis, preliminary evidence indicates that greater cellular peripheral immunoreactivity is associated with advanced brain age (Leech et al. 2025), suggesting that immune‐related processes may influence aging phenotypes. Future studies integrating neuroimaging with inflammatory biomarkers will be important for clarifying the relative contributions of these mechanisms.

Methodologically, this study illustrates the feasibility of training deep‐learning models exclusively on HC data to derive normative brain‐age estimates, thereby reducing potential influence of illness‐related features. While similar normative modeling approaches have been used in prior brain‐age studies (De Bonis et al. 2024; Group ECHRfPW et al. 2024; Rutherford et al. 2023), the present work applies this framework with post hoc interpretability via Grad–CAM to both SZ and BD within a harmonized, single‐site pipeline. The CNN showed strong performance in HCs, supporting internal consistency, although prediction errors were larger in patient groups. Interpretation of these results is therefore subject to several important considerations, which are discussed below.

Direct contrasts revealed no significant differences in mean Brain–PAD between BD and SZ. However, the associations between Brain–PAD and regional activation differed markedly between groups, with negative associations in BD and positive associations in SZ. These opposing patterns indicate that similar levels of apparent brain aging can be associated with distinct neural signatures across diagnostic categories, cautioning against interpretations of shared underlying mechanisms. Several factors may contribute to this counterintuitive pattern. First, survivor or selection bias may be at play, whereby individuals with more severe early disease burden or accelerated aging are underrepresented in older age groups. Second, differential age sampling and cohort effects across diagnostic groups may influence apparent trajectories in cross‐sectional analyses. Third, model‐related age bias cannot be excluded, as brain‐age prediction models are known to exhibit regression toward the mean age of the training set, particularly at the extremes of the age distribution. Importantly, these factors are not mutually exclusive. Given these considerations and the cross‐sectional design of the study, this late‐life pattern should not be interpreted as evidence of biological reversal or protection but rather as a limitation of inference that underscores the need for longitudinal, age‐balanced data sets. It should be noted that the modest size of the HC training and test samples represents an important limitation for deep learning–based brain‐age prediction and reported HC accuracy should be interpreted cautiously. Our primary aim was not to develop a maximally accurate brain‐age model, but to examine relative group differences using a consistent framework; nevertheless, future work using larger normative data sets and benchmarking against established models will be important for contextualizing performance.

An important consideration is that mean Brain–PAD group differences were modest relative to the prediction error observed in patient groups. While Brain–PAD differed significantly between groups at the population level, the larger MAE in BD and SZ limits individual‐level interpretability and underscores that Brain–PAD is best viewed as a noisy cross‐sectional phenotype rather than a precise estimate of individual brain age. This scale mismatch does not preclude group‐level inference but does increase uncertainty when modeling Brain–PAD as a function of age and may amplify sensitivity to residual bias. Although age‐bias correction based on HCs was applied prior to age‐trajectory analyses, prediction error and calibration limitations remain, particularly in clinical populations. Accordingly, age‐related Brain–PAD effects should be interpreted cautiously, and future studies employing larger normative data sets, alternative calibration strategies, and longitudinal designs will be essential for improving individual‐level precision and validating age‐trajectory findings.

Interpretation of post hoc Grad–CAM analyses warrants particular caution. While Grad–CAM highlights regions that contribute disproportionately to Brain–PAD predictions, these maps reflect sensitivity of the trained model rather than localized neurobiological mechanisms or causal anatomical changes. Accordingly, apparent regional emphasis should not be interpreted as evidence of focal pathology or focal aging processes, especially in the absence of robust region‐wise statistical significance after correction. Instead, the observed associations between Brain–PAD and CNN attention patterns, most prominently in frontal and temporal regions—likely reflect a combination of globally distributed structural deviation and regionally weighted model sensitivity to areas broadly vulnerable to normative age‐related or disease‐related change. As such, the present findings cannot distinguish between globally distorted neural aging and regionally specific biological mechanisms (e.g., neuroinflammatory effects) and should be interpreted as exploratory. Overall, Grad–CAM results are best viewed as illustrative of model behavior rather than as maps of biological aging.

As stated before, an additional consideration is the possibility of survivor bias. The apparent attenuation of Brain–PAD slopes in older BD and SZ participants may not reflect true normalization but rather selective survival, whereby individuals with the greatest illness burden or accelerated neurobiological aging are less likely to reach late adulthood or participate in research. Those remaining may represent a more resilient subgroup, consistent with epidemiological data showing life expectancy reductions of 10–20 years in SZ and BD (Walker et al. 2004; Chesney et al. 2014). This highlights the need for longitudinal and population‐based cohorts to disentangle true within‐person aging trajectories from selective survival effects.

It should also be noted that interpretation of Brain–PAD requires caution, particularly when discussing age‐related patterns. Brain–PAD derived from cross‐sectional prediction reflects deviation from age‐expected brain structure at a single time point and does not provide a direct estimate of the pace or rate of biological aging. Moreover, Brain–PAD is sensitive to age‐dependent prediction bias and calibration, especially at the extremes of the age distribution. Accordingly, terms such as “accelerated aging” should be understood in this study as shorthand for older‐appearing brain structure relative to chronological age, rather than evidence of increased longitudinal aging speed. Definitive assessment of aging rates will require longitudinal imaging with repeated within‐subject measurements.

Several limitations should be noted. First, the single‐site design represents an important constraint on external validity. While the use of a harmonized acquisition and processing pipeline reduces technical variability, it also limits generalizability across scanners, sites, and populations. Accordingly, findings regarding apparent brain aging and age‐related trajectories should be interpreted cautiously and viewed as preliminary. Replication in larger, multi‐site and longitudinal cohorts will be essential to establish robustness, clarify diagnosis‐specific versus overlapping effects, and determine the clinical relevance of brain‐age metrics across diverse samples. Second, an important limitation of this pilot study is the modest size of the healthy control (HC) cohort, particularly with respect to training and testing a deep learning–based brain‐age model. While the CNN achieved high accuracy in HCs and yielded consistent age‐by‐group effects across linear, spline, and piecewise models, absolute performance metrics should be interpreted cautiously, as smaller normative samples may limit model stability and inflate apparent accuracy. Moreover, the size of the HC cohort constrains the precision with which nonlinear normative brain‐aging trajectories, particularly spline‐ and piecewise‐based patterns, can be estimated and validated. As a result, the observed non‐uniform and biphasic Brain–PAD trajectories should be considered preliminary. Brain‐age models are known to exhibit age‐related biases, including regression toward the mean of the training distribution, particularly at the extremes of the age range. In combination with uneven age sampling and the limited number of older participants, these factors limit inference regarding late‐life divergence. Accordingly, the apparent convergence and renewed divergence observed in later adulthood should not be interpreted as evidence of true accelerated aging but rather as descriptive, hypothesis‐generating patterns that require longitudinal validation. Future studies leveraging larger, multi‐site normative data sets will be critical for training and validating nonlinear models of typical brain aging and for more precisely quantifying when and how patient trajectories diverge from normative patterns. Third, the sample included relatively few older adults across all groups, potentially reducing sensitivity to late‐life effects and limiting characterization of Brain–PAD trajectories in aging populations. Fourth, detailed medication histories were not available, particularly for BD, preventing direct evaluation of lithium or other treatment‐related influences on brain‐age estimates. An additional limitation is the absence of detailed, harmonized measures of medication exposure, illness duration, episode burden, and symptom severity in the primary analyses. These factors are likely to contribute to both between‐group differences and within‐group heterogeneity in Brain–PAD and may interact with age‐related effects. As such, apparent brain aging in the present study should be interpreted as an aggregate phenotype reflecting multiple, partially overlapping influences rather than a single mechanistic pathway. Future longitudinal studies incorporating detailed clinical trajectories and treatment histories will be necessary to clarify how disease burden and medication exposure shape brain aging across the lifespan. Finally, while Grad–CAM provided useful post hoc interpretability, its spatial resolution is limited, and complementary approaches (e.g., layer‐wise relevance propagation or SHAP) may offer more fine‐grained insights into regional contributions.

Despite these limitations, this pilot study provides a proof‐of‐concept for integrating CNN‐based brain‐age modeling with post hoc interpretability tools in psychiatric research. Within this single‐site cohort, the findings replicate the canonical rank order of apparent brain aging (SZ > BD > HC), reveal age‐dependent heterogeneity in Brain–PAD trajectories, and highlight frontotemporal regions as features to which the model is particularly sensitive when estimating brain age. More broadly, these results illustrate the potential utility of Brain–PAD as a quantitative phenotype for characterizing heterogeneity within and across severe mental illnesses. Future work in larger, multi‐site, and longitudinal cohorts incorporating detailed clinical information, such as illness duration, symptom severity, functional outcomes, and medication exposure will be essential to establish generalizability, disentangle biological from treatment‐related effects, and determine whether brain‐age metrics have prognostic or treatment‐relevant value.

## Funding

This work was supported by National Institutes of Health, R01MH114982, P50MH115846, R01MH095809, R01AG066670, R01MH135093, K23MH118565, K23MH121781.

## Conflicts of Interest

The authors declare no conflicts of interest.

## Supporting information


**Data S1:** hbm70479‐sup‐0001‐Supinfo.docx.

## Data Availability

The data that support the findings of this study are available on request from the corresponding author. The data are not publicly available due to privacy or ethical restrictions.
