# Supplementary material for: Deep Learning–Based Structural Brain Age Estimation in Bipolar Disorder and Schizophrenia: A Single‐Site Pilot Study
Source: Hum Brain Mapp. 2026 Feb 19;47(3):e70479. doi: 10.1002/hbm.70479 (PMC12920260; doi:10.1002/hbm.70479)
Supplement: Supplementary file 1 — Data S1: hbm70479‐sup‐0001‐Supinfo.docx. [file HBM-47-e70479-s001.docx]

**SUPPLEMENTARY MATERIAL**

**Supplementary Figure S01**

**
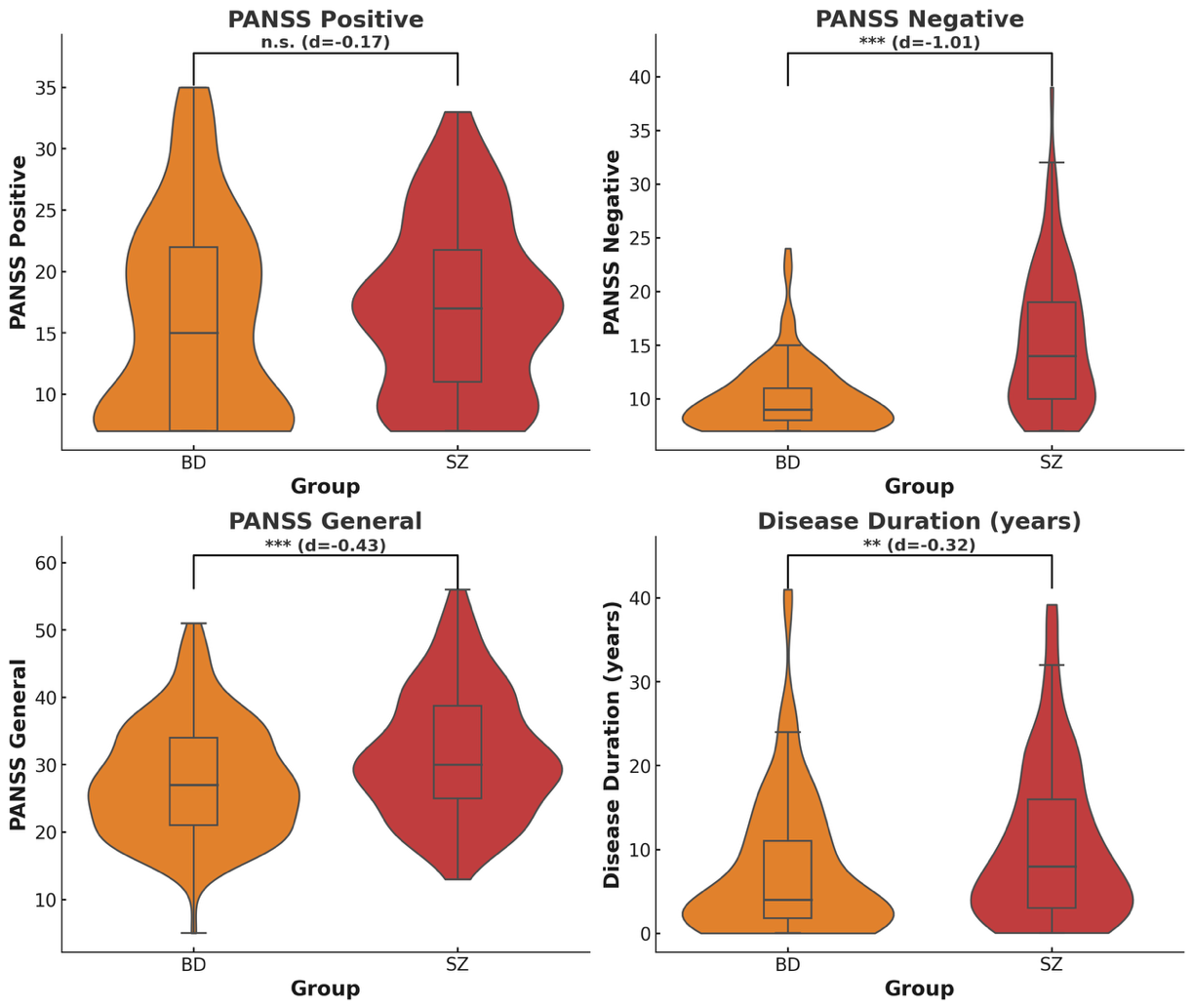
**

**Supplementary Figure S01. Distributions of PANSS symptom domains and disease duration in BD and SZ.** Violin plots show the distribution of scores for the Positive and Negative Syndrome Scale (PANSS) subscales (positive, negative, general) and disease duration (years) in patients with BD (orange) and SZ (red). Within each violin the embedded boxplots display the median and interquartile range. Horizontal brackets indicate between-group comparisons (BD vs SZ), with significance levels denoted as p < 0.01 (**), and p < 0.001 (***). Effect sizes are reported as Cohen’s d values.

**Supplementary Figure S02**

**
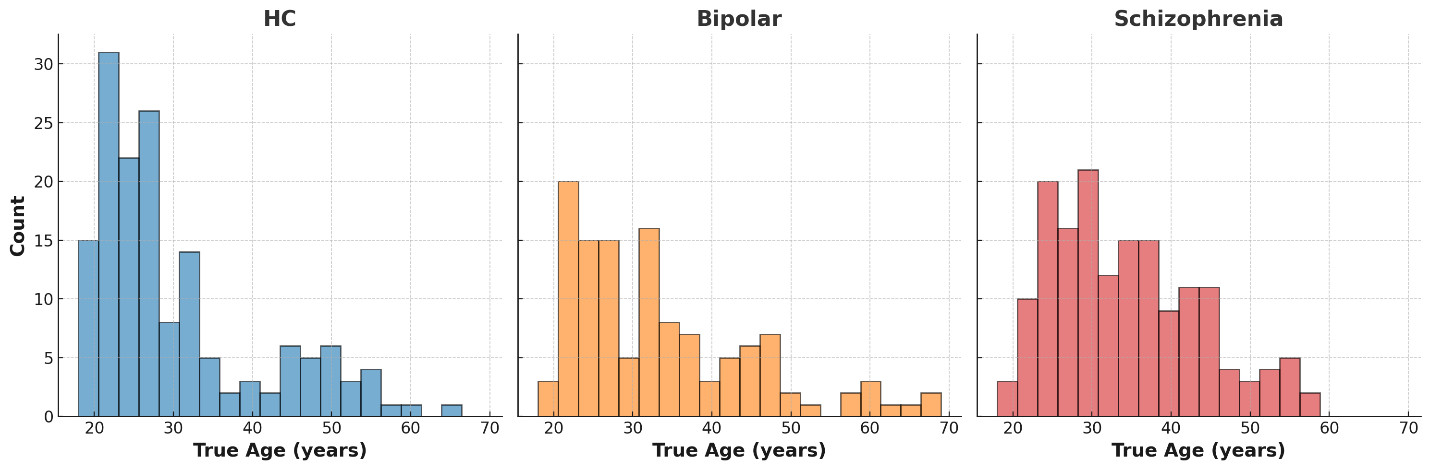
**

**Supplementary Figure S02. Age distributions across diagnostic groups**. Histograms of chronological age are shown separately for HCs, BD, and SZ. While all groups spanned early adulthood through late middle age, HCs were skewed toward younger ages, BD showed a broader distribution with fewer older individuals, and SZ clustered primarily in young-to-mid adulthood. These distributions contextualize age-by-group analyses of Brain-PAD trajectories presented in the main text.

**(Next Page)**

**Supplementary Figure S03**

**
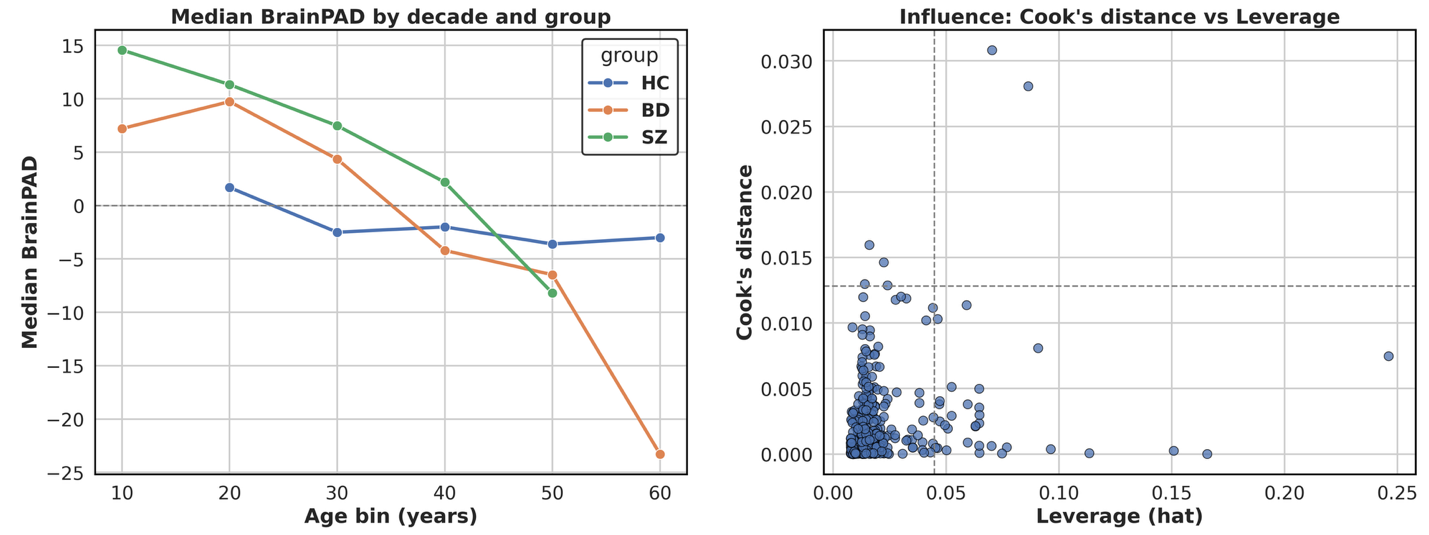
**

**Supplementary Figure S03. Age-stratified Brain-PAD trajectories and influence diagnostics.** (**Left**) Median Brain-PAD plotted by decade across groups (HC, BD, SZ). Both BD and SZ show increasingly negative trajectories with advancing age, particularly after midlife, while HC values remain near zero. (**Right**) Cook’s distance versus leverage plot for the linear interaction model. No participants exceeded conventional influence thresholds, indicating that results were not driven by outliers or high-leverage cases.

**Supplementary Table S1. Linear model testing age-by-group effects on Brain-PAD**

| **Predictor** | **β** | **SE** | **t** | **p** | **Interpretation** |
| --- | --- | --- | --- | --- | --- |
| Intercept (HC) | 4.15 | 3.52 | 1.18 | 0.240 | Baseline Brain-PAD at reference age (HC group) |
| Group: BD | +22.07 | 3.94 | 5.60 | <0.001 | BD shows higher Brain-PAD (older-appearing brains) vs HC at younger ages |
| Group: SZ | +23.87 | 4.00 | 5.96 | <0.001 | SZ shows higher Brain-PAD (older-appearing brains) vs HC at younger ages |
| True Age | –0.14 | 0.10 | –1.43 | 0.153 | Small, nonsignificant decline in Brain-PAD with age in HC |
| Group × Age (BD) | –0.51 | 0.11 | –4.63 | <0.001 | BD shows steeper Brain-PAD decline with age (accelerated trajectory) |
| Group × Age (SZ)  Sex(T.m]  Scanner Type [T.1] | –0.48  0.135  0.232 | 0.11  3.21  1.34 | –4.26  0.17  0.41 | <0.001  0.869  0.543 | SZ shows steeper Brain-PAD decline with age (accelerated trajectory)  Effect of scanner type 2 (Prisma) vs Magnetome |
| Model summary: | R² = 0.42 | Adj. R² = 0.41 | F(5, N–6) = 58.3 | p < 0.001 | Model explains 41% variance in Brain-PAD |
